# Quercetin, Kaempferol and Capsaicin Counteract the TGF-β1-Induced Upregulation of αSMA and Collagen in Myoblasts

**DOI:** 10.3390/ijms26115151

**Published:** 2025-05-27

**Authors:** Iris Cuijpers, Mireille M. J. P. E. Sthijns, Veerle A. R. van den Bogart, Joey Katsburg, Cliff F. M. Leenders, Freddy J. Troost

**Affiliations:** 1 Department of Human Biology, Institute of Nutrition and Translational Research in Metabolism (NUTRIM), Maastricht University, 6200 MD Maastricht, The Netherlands; 2 Food Innovation and Health, Centre for Healthy Eating and Food Innovation (HEFI), Maastricht University Campus Venlo, 5911 BV Venlo, The Netherlands

**Keywords:** quercetin, kaempferol, capsaicin, myofibroblast differentiation, myogenic differentiation, extracellular matrix

## Abstract

In fibrotic skeletal muscles, excessive extracellular matrix (ECM) deposition is a result of increased activation and decreased apoptosis of myofibroblasts. The aim of this study is to investigate whether treatment with quercetin, kaempferol or capsaicin can reduce the transforming growth factor-beta 1 (TGF-β1)-induced myofibroblast differentiation and fibrotic ECM expression in differentiated C2C12 cells. Two-day-differentiated C2C12 cells were treated with TGF-β1 for 48 h to induce myofibroblast differentiation. Twenty-four hours before (pre-treatment) and for forty-eight hours with (co-treatment) TGF-β1 treatment, cells were exposed to quercetin (25, 50 µM), kaempferol (10, 25, 50 µM) or capsaicin (25, 50 µM). The immunofluorescence intensity of alpha smooth muscle actin (αSMA) and collagen type I/III gene expression were assessed as myofibroblast markers. MyoD immunofluorescence intensity was measured as a myogenic marker. Co-treatment of TGF-β1 with the phytochemicals was most effective, resulting in a decreased number of αSMA-positive cells (all three compounds), decreased collagen type I (kaempferol, capsaicin) and type III (kaempferol) gene expression, and increased MyoD (kaempferol, capsaicin) protein expression compared to TGF-β1 treatment. This study demonstrates that treatment with quercetin, kaempferol or capsaicin can reduce myofibroblast markers. This suggests a possible anti-fibrotic effect of the phytochemicals in skeletal muscle.

## 1. Introduction

Aging is associated with a gradual decline in skeletal muscle mass and strength, a condition known as sarcopenia, and an increase in fibrotic muscle tissue. This leads to decreased physical function, which could potentially lead to disability, increased risk of nursing home admission and a higher mortality risk in older adults [1,2]. Between 2010 and 2023, sarcopenia affected 10 to 16% of the global elderly population [3]. The prevalence was even higher in specific patient groups, ranging from 18% in individuals with diabetes to 66% in patients with advanced esophageal cancer [3]. Furthermore, it has been estimated that the number of sarcopenic individuals in Europe will dramatically increase by 63.8 to 72.4% between 2016 and 2045 due to the increasing elderly population [4]. Aging skeletal muscles exhibit a reduced number of functional muscle stem cells and a diminished capacity to regenerate damaged muscle tissue compared to younger individuals [5,6]. Both intrinsic (e.g., changes in epigenetics, cellular senescence and apoptosis) and extrinsic changes, including increased local inflammation, contribute to the development of sarcopenia. Moreover, alterations in extracellular matrix (ECM) composition and growth factors within the muscle stem cell niche further impair skeletal muscle function [7]. Chronic local inflammation in aged skeletal muscle leads to an increased and persistent presence of transforming growth factor beta 1 (TGF-β1). Anti-inflammatory macrophages primarily mediate the release of TGF-β1, which promotes the differentiation of fibro-adipogenic progenitors, fibroblasts and myoblasts into myofibroblasts [8,9]. When TGF-β1 binds to its receptor, SMAD2/3 proteins are phosphorylated, forming a complex with SMAD4 that translocates to the nucleus. This SMAD complex functions as a transcription factor, upregulating the expression of alpha smooth muscle actin (αSMA), a contractile stress fiber abundantly present in myofibroblasts [8,10]. Activation of SMAD proteins also enhances gene expression of collagen type I (*Col1a1*), collagen type III (*Col3a1*) and other ECM components (such as fibronectin, laminin and proteoglycans) [11]. The secretion of ECM proteins by activated myofibroblasts results in structural remodeling of the ECM, which is crucial for both normal skeletal muscle regeneration and the development of skeletal muscle fibrosis [12]. While ECM remodeling is essential for optimal muscle regeneration and protection against further damage [13], chronic TGF-β1 release in aging skeletal muscle leads to prolonged myofibroblast activation, inhibition of myofibroblast apoptosis and excessive accumulation of collagen and other ECM components [14]. Mechanistically, TGF-β1 enhances reactive oxygen species (ROS) production by upregulating the expression of nicotinamide adenine dinucleotide phosphate oxidase (NADPH oxidase or Nox), impairing mitochondrial function and suppressing the antioxidant system [15,16,17]. Increased ROS production, in turn, enhances myofibroblast differentiation by activating latent TGF-β1 and stimulating TGF-β1 gene expression, creating a positive feedback loop that exacerbates collagen and other ECM components in skeletal muscle [18]. Fibrotic skeletal muscle tissue is formed that increases muscle stiffness and further impairs muscle stem cell function [19,20]. A large number of dietary phytochemicals have shown great potential in reducing fibrotic ECM expression in various cell types and organs. In vitro studies have shown that quercetin (a flavanol found in capers, onions and berries among others), kaempferol (present in capers, spinach, cabbage and broccoli) and capsaicin (the primary spicy compound in chili peppers) reduce fibrotic ECM expression in various cell types, including (cardiac/skin) fibroblasts, hepatic cells and bronchial and tubular epithelial cells [21,22,23,24,25,26,27,28,29]. Additionally, supplementation with these phyotchemicals has been reported to reduce fibrotic tissue formation in the kidney, liver, skin, lung and heart in murine models [22,23,24,25,26,27,28,29,30,31,32]. In addition to the mentioned effects on ECM, treatment with quercetin, kaempferol and capsaicin have been shown to decrease ROS and Nox2/4 protein or mRNA expression in different cell types and in mice models of skeletal muscle atrophy and skin fibrosis [28,33,34]. Additionally, treatment with quercetin and capsaicin in animal studies has been shown to protect against fibrosis via activation of the nuclear factor erythroid 2-related factor 2 (Nrf-2) pathway [30,35]. Nrf-2 is a key regulator of oxidative status and is responsible for the upregulation of genes encoding antioxidant enzymes (e.g., glutamate cysteine ligase catalytic subunit (GCLC), glutathione (GSH)) in response to ROS [36]. Despite the abundance of evidence showing the inhibitory effects of quercetin, kaempferol and capsaicin on fibrotic ECM expression in a wide range of cell types and organs, these effects and the influence of antioxidant pathways have not been shown in myoblast-derived myofibroblasts or in skeletal muscle.

The aim of the current study is to investigate whether treatment with the phytochemicals quercetin, kaempferol, or capsaicin attenuates TGF-β1-induced myofibroblast differentiation and fibrotic ECM expression in differentiating C2C12 cells. Additionally, the effects on cell apoptosis and the influence of Nox4 and GCLC are explored by assessing caspase-3 activity and *Nox4* and *GCLC* gene expression in differentiated C2C12 cells exposed to TGF-β1. It is hypothesized that quercetin, kaempferol and capsaicin reduce myofibroblast differentiation characterized by a decrease in αSMA protein expression and collagen type I and III gene expression in C2C12 cells exposed to TGF-β1. Furthermore, decreased *Nox4* gene expression and increased *GCLC* gene expression are expected in these cells due to treatment with the phytochemical compounds.

## 2. Results

### 2.1. Pre-Treatment with Quercetin, Kaempferol or Capsaicin Did Not Affect αSMA Intensity in Two-Day-Differentiated C2C12 Cells Exposed to TGF-β1

**Figure 1:**
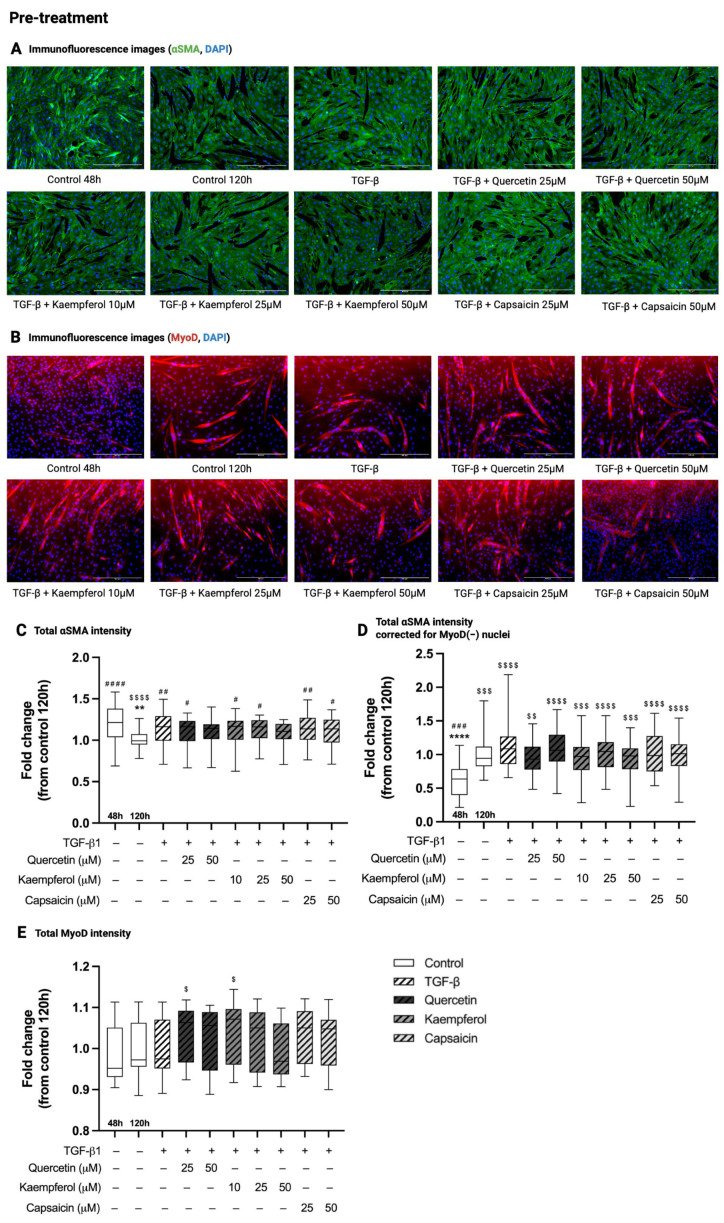
Effect of pre-treatments on alpha smooth muscle actin (αSMA) and MyoD immunofluorescence intensity. Immunofluorescence images (**A**,**B**) of two-day-differentiated C2C12 cells (control 48 h) treated with transforming growth factor beta 1 (TGF-β1, 5 ng/mL) for 48 h with and without 24 h pre-treatment with quercetin (25 and 50 µM), kaempferol (10, 25 and 50 µM) or capsaicin (25 and 50 µM). Cells were stained with alpha-smooth muscle actin (αSMA, green, **A**), myoblast determination protein 1 (MyoD, red, **B**) and 4′,6-Diamidino-2-phenylindole dihydrochloride (DAPI, blue, **A**, **B**) nuclei staining. In control cells at 120 h, total αSMA intensity (**C**) was decreased, total αSMA intensity corrected for the number of MyoD-negative (MyoD(–)) nuclei (**D**) was increased and MyoD intensity (**E**) was unchanged compared to control cells at 48 h of differentiation. Treatment with TGF-β1 increased total αSMA intensity (**C**), and did not alter total αSMA intensity corrected for the number of MyoD(–) nuclei (**D**) and MyoD intensity (**E**) compared to control 120 h. Pre-treatment of TGF-β1 with quercetin, kaempferol or capsaicin did not affect total αSMA intensity (**C**), total αSMA intensity corrected for the number of MyoD(-) nuclei (**D**) or MyoD intensity (**E**) compared to TGF-β1 treatment alone. ** *p* < 0.01; **** *p* < 0.0001, compared to TGF-β1. # *p* < 0.05; ## *p* < 0.01; #### *p* < 0.0001, compared to control 120 h. $ *p* < 0.05; $$ *p* < 0.01; $$$, *p* < 0.001, $$$$ *p* < 0.0001, compared to control 48 h. Scalebar = 400 µm. N = 3, *n* = 2, including 5 images per well. Created with Graphpad Prism 10.1.1 software (GraphPad Software, Boston, MA, USA) and BioRender.com (assessed on 26 of May, 2025).

Immunofluorescence images showing αSMA and MyoD intensity in all conditions of the pre-treatment experiment are shown in Figure 1A,B. In pre-treatment experiments, total αSMA immunofluorescence intensity was decreased in C2C12 control cells at 120 h compared to 48 h of differentiation (0.99 (0.95–1.07) versus 1.21 (1.04–1.38), *p* < 0.0001, Figure 1C). Treatment with TGF-β1 for 48 h in two-day-differentiated C2C12 cells increased total αSMA intensity compared to control cells at 120 h (1.16 (0.99–1.29) versus 0.99 (0.95–1.07), *p* = 0.0025, Figure 1C). Pre-treatment of two-day-differentiated C2C12 cells exposed to TGF-β1 with quercetin, kaempferol or capsaicin did not affect total αSMA intensity compared to TGF-β1 treatment alone (*p* > 0.99, Figure 1C). There was no effect of TGF-β1 (*p* > 0.99, compared to control 120 h), nor any of the pre-treatments (quercetin 25 and 50 µM, *p* = 0.40 and *p* > 0.99; kaempferol 10, 25 and 50 µM, *p* = 0.63, *p* > 0.99 and *p* = 0.62; capsaicin 25 and 50 µM, *p* > 0.99, respectively, compared to TGF-β1) on total αSMA intensity when corrected for the number of MyoD-negative nuclei (Figure 1D).

### 2.2. Pre-Treatment with Quercetin, Kaempferol or Capsaicin Did Not Affect MyoD Intensity in Two-Days Differentiated C2C12 Cells Exposed to TGF-β1

In pre-treatment experiments, there was no difference in total MyoD intensity between C2C12 control cells at 120 h and 48 h of differentiation (*p* > 0.99, Figure 1E). Treatment with TGF-β1 for 48 h in two-day-differentiated C2C12 did not affect total MyoD intensity compared to control cells at 120 h (*p* > 0.99, Figure 1E). Pre-treatment of two-day-differentiated C2C12 cells exposed to TGF-β1 with quercetin (25 and 50 µM; *p* = 0.47 and *p* > 0.99, respectively), kaempferol (10, 25 and 50 µM; *p* = 0.51, *p* > 0.99 and *p* > 0.99, respectively) or capsaicin (25 and 50 µM; *p* > 0.99) did not change total MyoD intensity compared to TGF-β1 treatment alone (Figure 1E).

### 2.3. Co-Treatment with Quercetin, Kaempferol or Capsaicin Reduced αSMA Intensity in Two-Day-Differentiated C2C12 Cells Exposed to TGF-β1

**Figure 2:**
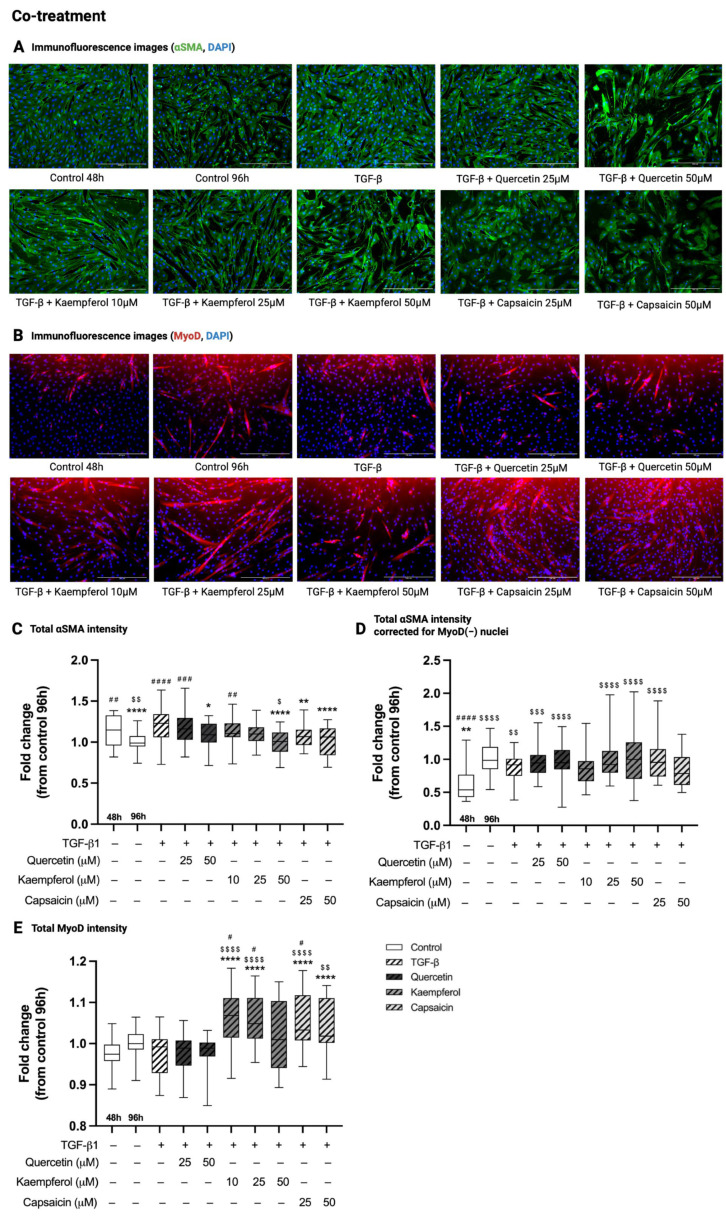
Effect of co-treatments on alpha smooth muscle actin (αSMA) and MyoD immunofluorescence intensity. Immunofluorescence images (**A**,**B**) of two-day-differentiated C2C12 cells (control 48 h) treated with transforming growth factor beta 1 (TGF-β1, 5 ng/mL) for 48 h with and without co-treatment with quercetin (25 and 50 µM), kaempferol (10, 25 and 50 µM) or capsaicin (25 and 50 µM). Cells were stained with alpha-smooth muscle actin (αSMA, green, **A**), myoblast determination protein 1 (MyoD, red, **B**) and 4′,6-Diamidino-2-phenylindole dihydrochloride (DAPI, blue, **A**,**B**) nuclear staining. In control cells, at 120 h, total αSMA intensity (**C**) was decreased, total αSMA intensity corrected for the number of MyoD-negative (MyoD(−)) nuclei (**D**) was increased, and MyoD intensity (**E**) was unchanged compared to control cells at 48 h of differentiation. Treatment with TGF-β1 increased total αSMA intensity (**C**) and did not alter total αSMA intensity corrected for the number of MyoD(−) nuclei (**D**) and MyoD intensity (**E**) compared to control 96 h. Co-treatment of TGF-β1 with quercetin, kaempferol, or capsaicin decreased total αSMA intensity (**C**) compared to TGF-β1 treatment alone, while the αSMA intensity was not affected when corrected for the number of MyoD-negative nuclei (**D**). Co-treatment with kaempferol or capsaicin increased the total intensity of MyoD compared to TGF-β1 treatment (**E**). * *p* < 0.05; ** *p* < 0.01; **** *p* < 0.0001, compared to TGF-β1. # *p* < 0.05; ## *p* < 0.01; ### *p* < 0.001; #### *p* < 0.0001, compared to control 96 h. $ *p* < 0.05; $$ *p* < 0.01; $$$ *p* < 0.001; $$$$ *p* < 0.0001, compared to control 48 h. Scalebar = 400 µm. N = 3, *n* = 2, including 5 images per well. Created with Graphpad Prism 10.1.1 software (GraphPad Software, Boston, MA, USA) and BioRender.com (assessed on 22 May 2025).

Immunofluorescence images showing αSMA and MyoD intensity in all conditions of the co-treatment experiment are shown in Figure 2A. In co-treatment experiments, total αSMA immunofluorescence intensity was decreased in C2C12 control cells at 96 h compared to 48 h of differentiation (0.99 (0.95–1.07) versus 1.33 (1.15–1.38), *p* = 0.0099, Figure 2C). Treatment with TGF-β1 for 48 h in two-day-differentiated C2C12 cells increased total αSMA intensity compared to control cells at 96 h (1.23 (1.06–1.34) versus 0.99 (0.95–1.07), *p* < 0.0001, Figure 2C). Co-treatment of two-day-differentiated C2C12 cells exposed to TGF-β1 with quercetin (50 µM, 1.09 (1.00–1.23), *p* = 0.025), kaempferol (50 µM, 1.01 (0.88–1.12), *p* < 0.0001) or capsaicin (25 and 50 µM, 1.07 (0.96–1.15) and 1.06 (0.84–1.17), *p* = 0.0051 and *p* < 0.0001, respectively) decreased total αSMA intensity compared to TGF-β1 treatment (1.23 (1.06–1.34)) alone (Figure 2C). However, there was no effect of TGF-β1, nor any of the co-treatments, when the total αSMA intensity was corrected for the number of MyoD-negative nuclei in these cells (*p* > 0.99, Figure 2D).

### 2.4. Co-Treatment with Kaempferol or Capsaicin Increased MyoD Intensity in Two-Day-Differentiated C2C12 Cells Exposed to TGF-β1

In co-treatment experiments, there was no difference in total MyoD intensity between C2C12 control cells at 96 h and 48 h of differentiation (*p* = 0.37, Figure 2E). Treatment with TGF-β1 for 48 h in two-day-differentiated C2C12 did not affect total MyoD intensity compared to control cells at 96 h (*p* > 0.99, Figure 2E). Co-treatment with quercetin (25 and 50 µM, *p* > 0.999, Figure 2E) did not change total MyoD intensity compared to TGF-β1. Co-treatment of TGF-β with kaempferol (10 and 25 µM, 1.07 (1.02–1.11) and 1.05 (1.01–1.11), *p* < 0.0001) or capsaicin (25 and 50 µM, 1.03 (1.01–1.12) and 1.02 (1.00–1.11), *p* < 0.0001 and *p* = 0.0059, respectively) increased total MyoD intensity compared to TGF-β1 treatment (0.99 (0.93–1.01), Figure 2E).

### 2.5. Pre-Treatment with Quercetin, Kaempferol or Capsaicin Did Not Affect the Number of Nuclei in Two-Day-Differentiated C2C12 Cells Exposed to TGF-β1

**Figure 3:**
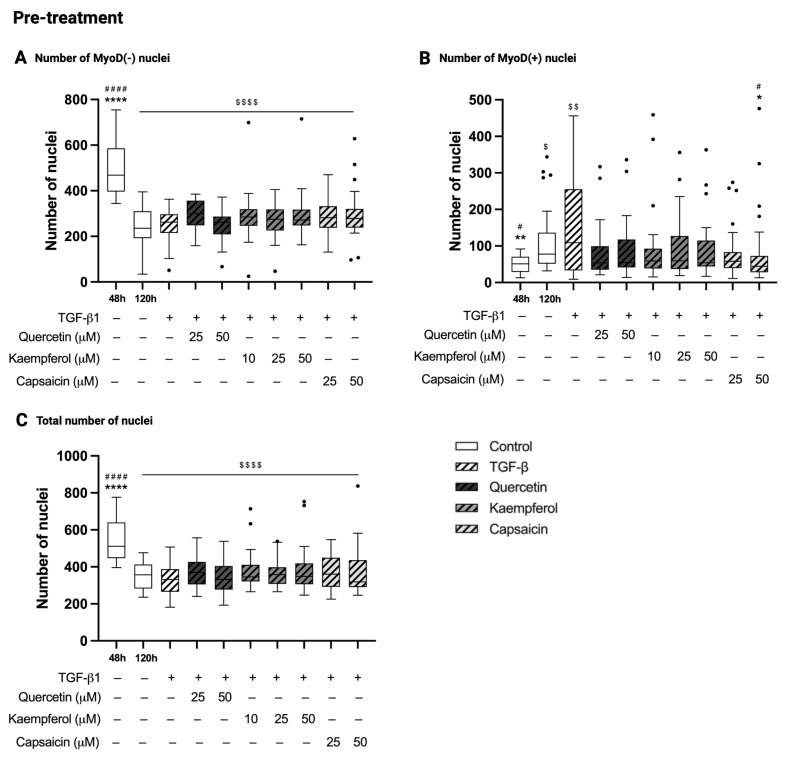
Effect of pre-treatments on the number of MyoD-negative, MyoD-positive and total nuclei. Number of MyoD-negative (MyoD(-)) nuclei (**A**), number of MyoD-positive (MyoD(+) nuclei (**B**) and total number of nuclei (**C**) of two-day-differentiated C2C12 cells (control 48 h) that are treated with transforming growth factor beta 1 (TGF-β1, 5 ng/mL) for 48 h with and without 24 h pre-treatment with quercetin (25 and 50 µM), kaempferol (10, 25 and 50 µM) or capsaicin (25 and 50 µM). Control cells at 120 h showed a decrease in the number of MyoD(−) nuclei (**A**), no change in the number of MyoD(+) nuclei (**B**) and a decrease in the total number of nuclei (**C**) compared to control cells at 48 h of differentiation. Treatment of 48 h differentiated C2C12 cells with TGF-β1 did not alter the number of MyoD(−) (**A**), the number of MyoD(+) (**B**) nor the total number of nuclei compared to control cell at 120 h. None of the pre-treatments did affect the number of MyoD(−) nuclei (**A**), MyoD(+) nuclei (**B**) or total number of nuclei (**C**), except for capsaicin in a concentration of 50 µM which decreased the number of MyoD(+) cells compared to TGF-β1 (**B**). ** *p* < 0.01; **** *p* < 0.0001, compared to TGF-β1. # *p* < 0.05; #### *p* < 0.0001, compared to control 96 h. $ *p* < 0.05; $$ *p* < 0.01; $$$$ *p* < 0.0001, compared to control 48 h. represent data points that are falling outside the whiskers of the boxplot. N = 3, *n* = 2, including 5 images per well. Created with Graphpad Prism 10.1.1 software (GraphPad Software, Boston, MA, USA) and BioRender.com (assessed on 22 May 2025).

In pre-treatment experiments, the number of MyoD-negative nuclei was decreased in C2C12 control cells at 120 h compared to 48 h of differentiation (236 (193–310) versus 469 (396–586), *p* < 0.0001, Figure 3A). The number of MyoD-positive nuclei was increased in C2C12 control cells at 120 h compared to 48 h of differentiation (78 (51–137) versus 51 (29–71), *p* = 0.012, Figure 3B). The number of total nuclei was decreased in C2C12 control cells at 120 h compared to 48 h of differentiation (357 (283–413) versus 511 (447–640), *p* < 0.0001, Figure 3C). Treatment with TGF-β1 for 48 h in two-day-differentiated C2C12 cells did not change the number of MyoD-negative nuclei (*p* > 0.99, Figure 3A), MyoD-positive nuclei (*p* > 0.99, Figure 3B), nor the total number of nuclei (*p* > 0.99, Figure 3C) compared to control cells at 120 h. Pre-treatment of two-day-differentiated C2C12 cells exposed to TGF-β1 with quercetin (25 and 50 µM) or kaempferol (10, 25 and 50 µM) did not change nuclei count of MyoD-negative cells (*p* = 0.44 for quercetin 25 µM and *p* > 0.99 for others, Figure 3A), MyoD-positive cells (*p* = 0.30 and *p* = 0.56, and *p* = 0.44, *p* = 0.81 and *p* = 0.61, respectively, Figure 3B), and the total number of nuclei (*p* = 0.95 and *p* > 0.99, and *p* = 0.96, *p* > 0.99 and *p* > 0.99, respectively, Figure 3C) compared to TGF-β1 treatment alone. Pre-treatment of two-day-differentiated C2C12 cells exposed to TGF-β1 with capsaicin (25 and 50 µM) did not affect the number of MyoD-negative nuclei (*p* > 0.99, Figure 3A) and total nuclei (*p* > 0.99, Figure 3C) compared to TGF-β1 treatment. The number of MyoD-positive cells was decreased by pre-treatment with capsaicin (50 µM, 45 (28–73) versus 109 (33–255), *p* = 0.011, Figure 3B), when compared to TGF-β1 treatment.

### 2.6. Co-Treatment with Kaempferol or Capsaicin Reduced the Number of MyoD-Negative Nuclei in Two-Day-Differentiated C2C12 Cells Exposed to TGF-β1

**Figure 4:**
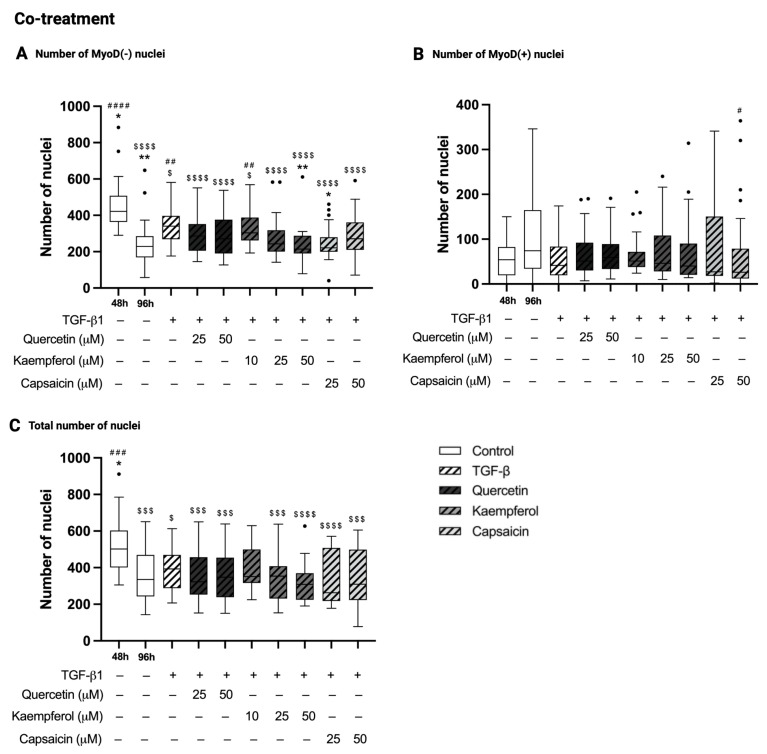
Effect of co-treatments on the number of MyoD-negative, MyoD-positive and total nuclei. Number of MyoD-negative (MyoD(−)) nuclei (**A**), number of MyoD-positive (MyoD(+) nuclei (**B**), and total number of nuclei (**C**) in two-day-differentiated C2C12 cells (control 48 h) that are treated with transforming growth factor beta 1 (TGF-β1, 5 ng/mL) for 48 h with and without co-treatment with quercetin (25 and 50 µM), kaempferol (10, 25 or 50 µM) or capsaicin (25 and 50 µM). Control cells at 96 h showed a decreased number of MyoD(−) nuclei (**A**), no difference in the number of MyoD(+) nuclei (**B**) and a decreased total nuclei count (**C**) compared to control cells at 48 h of differentiation. Treatment of 48 h differentiated C2C12 cells with TGF-β1 increased the number of MyoD(−) nuclei (**A**), and did not affect the number of MyoD(+) (**B**) and the total number of nuclei (**C**) compared to control 96 h. Co-treatment of TGF-β1 with quercetin did not affect the nuclei count compared to TGF-β1 treatment alone (**A**–**C**). The number of MyoD(−) nuclei was decreased (**A**), and the number of MyoD(+) (**B**) and total nuclei (**C**) were not affected by co-treatment of TGF-β1 with kaempferol or capsaicin when compared to TGF-β1 treatment alone. * *p* < 0.05; ** *p* < 0.01, compared to TGF-β1. # *p* < 0.05; ## *p* < 0.01; ### *p* < 0.001; #### *p* < 0.0001, compared to control 96 h. $ *p* < 0.05; $$$ *p* < 0.001; $$$$ *p* < 0.0001, compared to the control 48 h. • represent data points that are falling outside the whiskers of the boxplot. N = 3, *n* = 2, including 5 images per well. Created with Graphpad Prism 10.1.1 software (GraphPad Software, Boston, MA, USA) and BioRender.com (assessed on 22 May 2025).

In co-treatment experiments, the number of MyoD-negative nuclei was decreased in C2C12 control cells at 96 h compared to 48 h of differentiation (229 (169–285) versus 422 (364–508), *p* < 0.0001, Figure 4A). The number of MyoD-positive nuclei was similar in C2C12 control cells at 96 h compared to 48 h of differentiation (*p* = 0.46, Figure 4B). The total number of nuclei was diminished in C2C12 control cells at 96 h compared to 48 h of differentiation (335 (243–470) versus 502 (401–603), *p* = 0.0003, Figure 4C). Treatment with TGF-β1 for 48 h in two-day-differentiated C2C12 increased the number of MyoD-negative nuclei (340 (269–397 versus 229 (169–285), *p* = 0.017, Figure 4A), and did not alter the number of MyoD-positive nuclei (*p* = 0.26, Figure 4B) and total number of nuclei (*p* > 0.99, Figure 4C) compared to C2C12 control cells at 96 h. Co-treatment of two-day-differentiated C2C12 cells exposed to TGF-β1 with quercetin (25 and 50 µM) did not change nuclei count of MyoD-negative cells (*p* > 0.99 and *p* = 0.52, respectively, Figure 4A), MyoD-positive cells (*p* > 0.99 and *p* > 0.99, respectively, Figure 4B), nor the total number of nuclei (*p* < 0.99 and *p* > 0.99, respectively, Figure 4C) compared to treatment with TGF-β1 alone. Co-treatment of two-day-differentiated C2C12 cells exposed to TGF-β1 with kaempferol (50 µM) decreased the number of MyoD-negative nuclei (215 (191–288) versus 340 (269–397), *p* = 0.0026, Figure 4A), while the number of MyoD-positive nuclei (*p* > 0.99, Figure 4B) and the total number of nuclei (*p* = 0.16, Figure 4C) were not affected compared to treatment with TGF-β1 alone. A similar effect was seen for capsaicin in a concentration of 25 µM (decreased number of MyoD-negative nuclei 221 (199–280) versus 340 (269–397), *p* = 0.013, Figure 4A; no change in MyoD-positive nuclei *p* > 0.99, Figure 4B and total number of nuclei, *p* = 0.96, Figure 4C) compared to treatment with TGF-β1. For all immunohistochemistry outcomes of pre-treatment and co-treatment set-ups, the influence of solvent DMSO was tested, and did not show any significant differences compared to control 96 h and 120 h.

### 2.7. Co-Treatment with Kaempferol or Capsaicin Did Not Affect Caspase-3 Activity in Two-Day-Differentiated C2C12 Cells Exposed to TGF-β1

**Figure 5:**
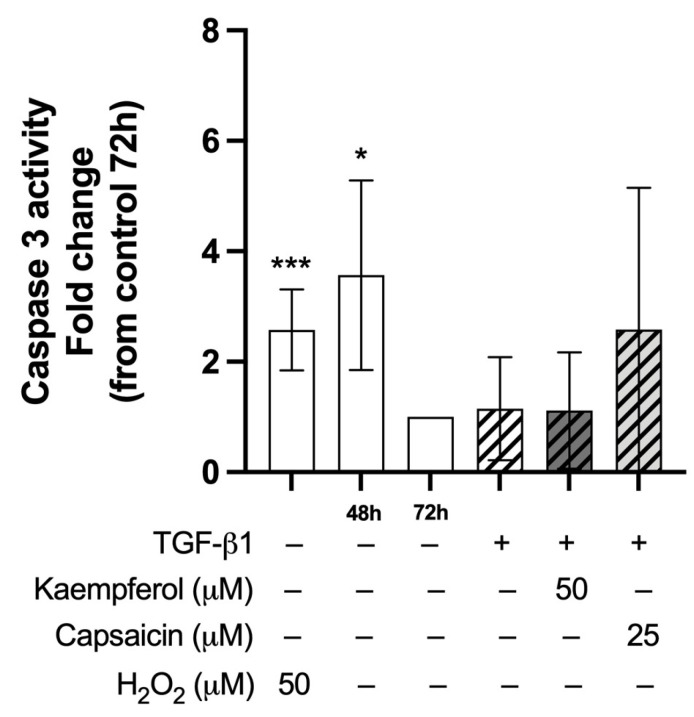
Effect of co-treatment with kaempferol and capsaicin on caspase-3 activity. Caspase-3 activity in two-day differentiated C2C12 cells (control 48 h) treated with transforming growth factor beta 1 (TGF-β1, 5 ng/mL) for 24 h with and without co-treatment with kaempferol (50 µM) or capsaicin (25 µM). Treatment with 50 µM hydrogen peroxide (H_2_O_2_) was used as a positive control and increased caspase-3 activity compared to the control (72 h). Control cells at 48 h of differentiation showed increased caspase activity compared to control cells 72 h. Neither treatment with TGF-β1 nor any of the co-treatments affected caspase-3 activity more than the control at 72 h. An unpaired T-test was used to test for a significant difference between the positive control condition and control (72 h). * *p* < 0.05; *** *p* < 0.001, compared to control 72 h. N = 3, *n* = 2, sample of two biological duplicates was pooled for caspase-3 analysis. Created with Graphpad Prism 10.1.1 software (GraphPad Software, Boston, MA, USA).

To determine whether differences in the number of nuclei could be explained by caspase-3-mediated apoptosis, the effects of TGF-β1 and co-treatment with kaempferol 50 µM or capsaicin 25 µM on caspase-3 activity were assessed (Figure 5). Treatment with 50 µM H_2_O_2_ increased caspase-3 activity compared to control cells at 72 h (2.58 ± 0.42, *p* < 0.001). Caspase-3 activity was increased in C2C12 control cells at 48 h of differentiation compared to control cells at 72 h (3.57 ± 1.77, *p* = 0.024). Treatment of 48 h differentiated C2C12 cells with TGF-β1 with or without kaempferol or capsaicin did not affect caspase-3 activity compared to control 72 h.

### 2.8. Pre-Treatment with Quercetin Reduced Collagen Type 1 (Col1a1) Gene Expression in Two-Day-Differentiated C2C12 Cells Exposed to TGF-β1

**Figure 6:**
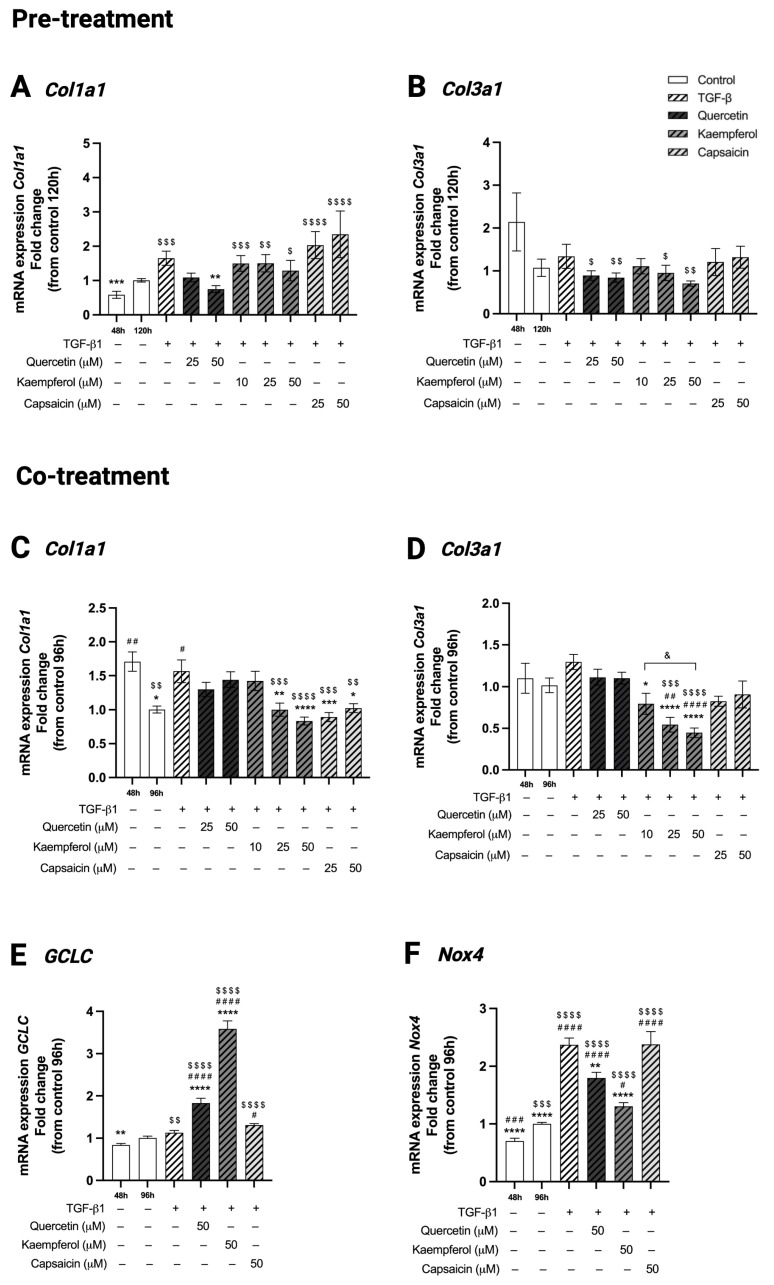
Effect of pre-treatments and co-treatments on gene expression of collagen, glutamate-cysteine ligase catalytic subunit and NADPH oxidase 4. Gene expression of collagen type I (*Col1a1*), collagen type III (*Col3a1*) and glutamate-cysteine ligase catalytic subunit (*GCLC*) assessed in two-day-differentiated C2C12 cells (control 48 h) treated with transforming growth factor beta 1 (TGF-β1, 5 ng/mL) for 48 h with and without 24 h pre-treatment or 48 h co-treatment with quercetin (25 or 50 µM), kaempferol (10, 25 or 50 µM) or capsaicin (25 or 50 µM). *Col1a1* and *Col3a1* gene expression was comparable between control cells at 120h and at 48h of differentiation (**A**,**B**). Treatment of 48h differentiated C2C12 cells with TGF-β1 did not affect *Col1a1* and *Col3a1* gene expression compared to control 120h (**A**,**B**). Pre-treatment of TGF-β1 with quercetin (50 µM) decreased *Col1a1* gene expression compared to TGF-β1 treatment alone (**A**). In pre-treatment experiments, none of the compounds induced an effect on *Col3a1* gene expression compared to TGF-β1 (**B**). In control cells at 96h, *Col1a1* gene expression was decreased (**C**), gene expression of *Col3a1* (**D**) and *GCLC* (**E**) was unchanged, and gene expression of *Nox4* was increased (**F**) compared to control cells at 48 of differentiation. Treatment of 48h differentiated C2C12 cells with TGF-β1 increased *Col1a1* (**C**) and *Nox4* (**F**) gene expression, and did not affect gene expression of *Col3a1* (**D**) and *GCLC* (**E**) compared to control cells at 96h. Co-treatment of TGF-β1 with kaempferol (25 and 50 µM) or capsaicin (25 and 50 µM) decreased *Col1a1* gene expression compared to TGF-β1 treatment alone (**C**). Co-treatment with kaempferol (10, 25 and 50 µM) decreased *Col3a1* gene expression compared to TGF-β1 treatment (**D**). Co-treatment with quercetin or kaempferol (both 50 µM) increased gene expression of *GCLC* compared to TGF-β1 treatment (**E**). Co-treatment with quercetin or kaempferol (both 50 µM) decreased *Nox4* gene expression compared to TGF-β1 treatment (**F**). * *p* < 0.05; ** *p* < 0.01; *** *p* < 0.001; **** *p* < 0.0001, compared to TGF-β1. # *p* < 0.05; ## *p* < 0.01; ### *p* < 0.001; #### *p* < 0.0001, compared to control 96/120h. $ *p* < 0.05; $$ *p* < 0.01; $$$ *p* < 0.001; $$$$ *p* < 0.0001, compared to control 48 h. & *p* < 0.05, between indicated conditions. N *=* 3, *n =* 2; each biological duplicate represents the mean of three technical replicates. Created with Graphpad Prism 10.1.1 software (GraphPad Software, Boston, MA, USA) and BioRender.com (assessed on 22 May 2025).

In pre-treatment experiments, gene expression of *Col1a1* and *Col3a1* was comparable between C2C12 control cells at 120 h and 48 h of differentiation (*p* = 0.14 and *p* = 0.34, respectively, Figure 6A,B). Treatment of two-day-differentiated C2C12 cells with TGF-β1 for 48 h did not affect *Col1a1* nor *Col3a1* gene expression compared to control cells at 120 h (*p* = 0.33 and *p* > 0.99, respectively, Figure 6A,B). Pre-treatment of two-day-differentiated C2C12 cells exposed to TGF-β1 with quercetin (50 µM) decreased *Col1a1* gene expression compared to treatment with TGF-β1 alone (1.74 ± 0.10 versus 1.65 ± 0.21, *p* = 0.0073, Figure 6A), while *Col3a1* expression was not affected (*p* > 0.99, Figure 6A,B). Pre-treatment of two-day-differentiated C2C12 cells exposed to TGF-β1 with kaempferol (10, 25 and 50 µM) as well as capsaicin (25 and 50 µM) did not change the gene expression of both *Col1a1* (*p* > 0.99, *p* = 0.99, *p* = 0.58, *p* = 0.98, *p* = 0.96, respectively, Figure 6A) or *Col3a1* (*p* > 0.99, *p* > 0.99, *p* = 0.41, *p* > 0.99, *p* > 0.99, respectively, Figure 6B) when compared to TGF-β1 treatment.

### 2.9. Co-Treatment with Kaempferol or Capsaicin Reduced Collagen Type I (Col1a1) and Kaempferol Reduced Collagen Type 3 (Col3a1) Gene Expression in Two-Day-Differentiated C2C12 Cells Exposed to TGF-β1

In co-treatment experiments, C2C12 control cells at 96 h showed a decreased *Col1a1* gene expression (1.00 ± 0.05 versus 1.71 ± 0.14, *p* = 0.0022, Figure 6C) compared to control cells at 48 h of differentiation, while *Col3a*1 expression (*p* > 0.99, Figure 6D) was comparable in control cells between the two time points. TGF-β1 treatment for 48 h in two-day-differentiated C2C12 cells increased *Col1a1* gene expression (1.57 ± 0.17 versus 1.00 ± 0.05, *p* = 0.01, Figure 6C) but did not affect *Col3a1* gene expression (*p* = 0.65, Figure 6D) compared to control 96 h. Co-treatment of two-day-differentiated C2C12 cells exposed to TGF-β1 with quercetin (25 and 50 µM) did not affect *Col1a1* (*p* = 0.66 and *p* = 0.99, respectively, Figure 6C) nor *Col3a1* gene expression (*p* = 0.93, Figure 6D) compared to TGF-β1 treatment alone. Co-treatment of two-day-differentiated C2C12 cells exposed to TGF-β1 with kaempferol decreased both *Col1a1* (25 and 50 µM, 1.00 ± 0.096 and 0.84 ± 0.06 versus 1.57 ± 0.17, *p* = 0.0047 and *p* < 0.0001, respectively, Figure 6C) and *Col3a1* gene expression (10, 25 and 50 µM, 0.80 ± 0.13, 0.54 ± 0.09 and 0.45 ± 0.06 versus 1.30 ± 0.09, *p* = 0.012, *p* < 0.0001 and *p* < 0.0001, respectively, Figure 6D) compared to TGF-β1 treatment, in a dose-dependent manner. In addition, there was a significant difference in *Col3a1* gene expression between kaempferol concentrations 10µM and 50 µM (*p* = 0.038), with the highest concentration being more effective compared to the lowest concentration. Co-treatment of two-day-differentiated C2C12 cells exposed to TGF-β1 with capsaicin (25 and 50 µM) decreased *Col1a1* gene expression (0.89 ± 0.07 and 1.03 ± 0.07 versus 1.57 ± 0.17, *p* = 0.0007 and *p* = 0.035, respectively, Figure 6C) but did not affect *Col3a1* gene expression (*p* = 0.08 and *p* = 0.22, respectively, Figure 6D) compared to TGF-β1 treatment. There was no effect of solvent DMSO on *Col1a1* and *Col3a1* gene expression compared to control cells at 96 and 120 h in both pre-treatment and co-treatment set-ups.

### 2.10. Co-Treatment with Quercetin or Kaempferol Increased GCLC Gene Expression in Two-Day-Differentiated C2C12 Cells Exposed to TGF-β1

In co-treatment experiments, *GCLC* gene expression was comparable between C2C12 control cells at 96 h and 48 h of differentiation (*p* = 0.096, Figure 6E). Treatment of two-day-differentiated C2C12 cells with TGF-β1 treatment for 48 h did not affect *GCLC* gene expression compared to control at 96 h (*p* = 0.40, Figure 6E). Co-treatment of two-day-differentiated C2C12 cells exposed to TGF-β1 with quercetin (1.83 ± 0.11 versus 1.00 ± 0.04, *p* < 0.0001, Figure 6E) or kaempferol (3.59 ± 0.19 versus 1.00 ± 0.04, *p* < 0.0001, Figure 6E), both at a concentration of 50 µM, increased *GCLC* gene expression compared to TGF-β1 treatment alone. Co-treatment of two-day-differentiated C2C12 cells exposed to TGF-β1 with capsaicin did not affect *GCLC* gene expression compared to TGF-β1 (*p* = 0.21, Figure 6E).

### 2.11. Co-Treatment with Quercetin or Kaempferol Decreased Nox4 Gene Expression in Two-Day-Differentiated C2C12 Cells Exposed to TGF-β1

In co-treatment experiments, *Nox4* gene expression was increased in control cells at 96 h compared to cells at 48 h of differentiation (1.00 ± 0.03 versus 0.70 ± 0.05, *p* = 0.0008, Figure 6F). TGF-β1 treatment for 48 h in two-day-differentiated C2C12 cells upregulated *Nox4* gene expression (2.37 ± 0.12 versus 1.00 ± 0.03, *p* = 0.01, Figure 6F) compared to control 96 h. Co-treatment of TGF-β1 with quercetin (50 µM, 1.80 ± 0.10 versus 2.37 ± 0.12, *p* = 0.0051) or kaempferol (50 µM, 1.31 ± 0.06 versus 2.37 ± 0.12, *p* < 0.0001) in two-day-differentiated C2C12 cells decreased *Nox4* gene expression compared to TGF-β1 treatment alone (Figure 6F). Co-treatment of TGF-β1 with 50 µM capsaicin did not alter the expression of *Nox4* compared to TGF-β1 treatment (*p* > 0.99, Figure 6F).

## 3. Discussion

To our knowledge, this is the first study investigating the effects of phytochemicals quercetin, kaempferol or capsaicin on myofibroblast differentiation in skeletal muscle derived cells. The degree of myofibroblast differentiation was determined via assessment of three myofibroblast markers (αSMA protein expression, collagen type I and III gene expression) and a myogenic marker (MyoD protein expression). Treatment of two-day-differentiated C2C12 cells with TGF-β1 for 48 h increased myofibroblast markers (total αSMA protein expression, collagen type I gene expression), and did not affect the expression of the myogenic marker MyoD. While pre-treatment strategies are often used to modulate signaling pathways before exposure to a fibrotic insult, pre-treatment with phytochemicals prior to TGF-β1 stimulation was not successful in diminishing the effects induced by TGF-β1. With the exception of pre-treatment with 50 µM quercetin, which decreased collagen type 1 gene expression compared to TGF-β1 treatment alone. Co-treatments were consistently more effective in reducing myofibroblast markers and increasing myogenic marker MyoD in C2C12 cells that were exposed to TGF-β1. Co-treatment of two-day-differentiated C2C12 cells exposed to TGF-β1 with quercetin, kaempferol and capsaicin, decreased total intensity of αSMA compared to TGF-β1 treatment alone. Co-treatment with kaempferol or capsaicin also decreased collagen type I gene expression and increased the expression of MyoD compared to TGF-β1 treatment. Additionally, co-treatment with kaempferol decreased collagen type III gene expression in two-day-differentiated C2C12 cells exposed to TGF-β1. 

It has been demonstrated that treatment with TGF-β1 stimulates fibrotic protein and gene expression in myogenic cells in vitro [37,38]. This is in agreement with our results, which showed increased αSMA protein expression and collagen type I gene expression when two-day-differentiated C2C12 were exposed to TGF-β1 for 48 h. TGF-β1 exposure did not affect αSMA expression when corrected for the number of MyoD-negative (but αSMA-positive) nuclei. This suggests that treatment with TGF-β1 resulted in an increase in the number of MyoD-negative (but αSMA-positive) cells, but that the expression of αSMA in one individual cell was not affected. In addition to stimulating myofibroblast markers, TGF-β1 has been shown to inhibit myogenic differentiation in C2C12 myoblasts via SMAD3-mediated repression of transcription factors belonging to the MyoD family [39]. However, treatment with TGF-β1 did not result in down-regulation of the myogenic marker MyoD in this study. This might be explained by the differentiation status of the cells. Previous studies have shown that once myoblasts commit to differentiation, they become insensitive to the inhibitory effect of TGF-β on myogenesis [40]. This could explain why TGF-β1 treatment did not affect MyoD expression in two-day-differentiated C2C12 cells in this study. 

Pre-treatment of TGF-β1 with the phytochemicals did not succeed in diminishing the effects induced by TGF-β1, with one exception being pre-treatment with 50 µM quercetin, which decreased collagen type 1 gene expression compared to TGF-β1 treatment. Other studies found anti-fibrotic effects of quercetin pre-treatment as well. In lung-derived fibroblasts, 3 h pre-treatment with quercetin (5–20 µM) decreased lipopolysaccharide (LPS)-induced mRNA expression of collagen type I [41]. In kidney fibroblasts, pre-treatment with 10 and 20 µM of quercetin for 30 min decreased TGF-β1-induced protein expression of αSMA [27]. 

Co-treatment with quercetin, kaempferol or capsaicin diminished the effects of TGF-β1 on myofibroblast differentiation in two-day-differentiated C2C12 cells. The main underlying mechanisms described for the inhibitory effects of these compounds on fibrotic ECM expression utilize interference with the TGF-β1 pathway, possibly by inhibiting SMAD2/3 phosphorylation either directly or via competitive binding on the kinase domain of the TGF-β type I receptor [29,32,42]. These observations were carried out in hepatic stellate cells, dermal fibroblasts, mouse heart tissue and in molecular docking studies and it would be interesting to investigate the influence of the SMAD pathway on the anti-fibrotic effects of the compounds in skeletal muscle-derived cells or tissue in future studies. In addition, phytochemicals may inhibit TGF-β1 pathway activation via their antioxidant activity. In a wide range of cell types, it has been shown that TGF-β1 increases ROS production by upregulating the expression of Nox, impairing mitochondrial function and suppressing the antioxidant system [15,16,17]. In turn, increased ROS production enhances myofibroblast differentiation by activating latent TGF-β1 and increasing TGF-β1 gene expression, creating a positive feedback loop [18]. Treatment with quercetin, kaempferol or capsaicin has been shown to decrease ROS and Nox2/4 protein or gene expression in different cell types and in mice models of skeletal muscle atrophy and skin fibrosis [28,33,34]. This is in accordance with the results from this study showing that co-treatment with quercetin or kaempferol decreased the TGF-β1-induced gene expression of *Nox4* in two-day-differentiated C2C12 cells. However, co-treatment of TGF-β1 with capsaicin did not change *Nox4* gene expression compared to TGF-β1 treatment alone. Additionally, the phytochemicals could protect against ROS via upregulation of Nrf-2. Nrf-2 is a key regulator of oxidative status and is responsible for the upregulation of genes encoding antioxidant enzymes (e.g., GCLC, GSH) in response to ROS [36]. Treatment of TGF-β1 exposed kidney fibroblasts with quercetin increased intracellular Nrf-2 levels and promoted its nuclear translocation [35]. The activation of Nrf-2 was suggested to be involved in the anti-fibrotic effects of quercetin, as Nrf-2 knockdown suppressed the inhibitory effects of quercetin on fibroblast activation and collagen deposition in these cells [35]. Treatment with kaempferol has also been shown to activate the Nrf-2 signaling pathway and increase the expression of GCLC in hepatocytes and mouse auditory cells [43,44]. This is in line with the upregulation of *GCLC* gene expression shown in TGF-β1 exposed cells that were co-treated with quercetin or kaempferol in this study. Capsaicin co-treatment did not influence *GCLC* gene expression. Together, these results suggest that quercetin and kaempferol exert antifibrotic effects via antioxidant pathways, including GCLC stimulation and Nox4 inhibition. These mechanisms could be further validated through gene knockdown experiments targeting Nox4 or antioxidant genes in C2C12 cells. 

Surprisingly, αSMA intensity was not affected by the co-treatments when corrected for the number of MyoD-negative (but αSMA-positive) nuclei. This suggests that co-treatments did not affect the αSMA expression in individual cells, but instead reduced the number of αSMA-expressing cells. Our results showed that co-treatment of two-day-differentiated C2C12 cells exposed to TGF-β1 with kaempferol or capsaicin decreased the number of MyoD-negative nuclei, while the number of MyoD-positive nuclei and total nuclei was not affected compared to treatment with TGF-β1. This may indicate a pro-apoptotic effect of the phytochemicals on cells that express αSMA but not MyoD. TGF-β1 has been shown to suppress the expression of apoptotic protein BCL-2-like protein 4 (BAX) and to enhance the expression of anti-apoptosis regulator B-cell lymphoma 2 (BCL-2). A reduction in the BAX:BCL-2 ratio inhibits caspase-3-mediated myofibroblast apoptosis [45]. Inhibition of myofibroblast apoptosis by TGF-β1 results in persistent activation and production of pro-fibrotic proteins [46]. Therefore, stimulation of myofibroblast apoptosis by the phytochemicals may avoid the evasion of apoptosis induced by TGF-β1. Despite observed differences in the number of nuclei, neither TGF-β1 nor any of the co-treatments affected caspase-3 activity compared to the control or compared to TGF-β1 treatment, respectively. Furthermore, there was no indication of cytotoxicity after 48 h treatment of TGF-β1 and after 24 h treatment with the phytochemical compounds assessed with a lactate dehydrogenase (LDH) assay. 

Our findings indicate that the phytochemicals exhibit distinct effects and timing profiles, suggesting different mechanisms of action. Co-treatment was more effective than pre-treatment in reducing total αSMA intensity for all three phytochemicals. The duration of exposure may explain these differences; pre-treatment lasted 24 h, while co-treatments were applied for 48 h, concurrently with TGF-β1. Furthermore, TGF-β1 rapidly upregulates its own receptor at the cell surface, amplifying its effects [47]. If phytochemicals inhibit this signaling, simultaneous exposure may be more effective than pre-treatment. Interestingly, while pre-treatment with quercetin reduced collagen gene expression, co-treatment did not. This is in contrast with the effects of kaempferol and capsaicin, which induced a decrease in collagen expression in the co-treatment set-up. Our data indicate that treatment with quercetin results in the stimulation of an alternative molecular pathway that induces its additional effectiveness as a pre-treatment. A hypothesis for this is that quercetin has a higher potential to upregulate antioxidant enzymes (such as GCLC in combination with other Nrf-2 mediated antioxidants) compared to kaempferol and capsaicin. Increased levels of intracellular antioxidants before stimulation with TGF-β1 could reduce the exacerbating effects of ROS on the TGF-β1 pathway. The high number of free hydroxyl groups in the chemical structure of quercetin could indicate an increased antioxidant capacity compared to other phytochemicals [48,49]. However, the potential of quercetin to upregulate intracellular antioxidant enzymes compared to others should be investigated further. Other studies showed that the combined inhibition of the mammalian target of rapamycin (mTOR) and catenin beta-1 (β-catenin) signaling might be involved in the anti-fibrotic effects of quercetin [27,41].

In addition to the effects of the phytochemicals in reducing myofibroblast markers, co-treatment of TGF-β with kaempferol or capsaicin also increased protein expression of myogenic marker MyoD. Studies investigating the myogenic potential of kaempferol or capsaicin and their underlying mechanisms are limited. Kaempferol treatment in C2C12 cells has been shown to increase the expression of myosin heavy chain through activation of integrin-β1/focal adhesion kinase/paxillin (ITG1B/FAK/paxillin) and insulin-like growth factor 1/protein kinase B/mTOR (IGF1R/AKT/mTOR) pathways [50]. Other plant bioactives, including hesperetin, dehydrocorydaline and curcumin, have been shown to enhance C2C12 differentiation via increased localization and myogenin promotor binding of MyoD, via p38 mitogen-activated protein kinase (MAPK) activation or increased Wnt signaling, respectively [51,52,53]. More research is necessary to elucidate the underlying mechanisms of the stimulating effects of kaempferol and capsaicin on MyoD expression found in this study. 

In addition to pre-treatment and co-treatment set-ups, it would be interesting to investigate the effects of the phytochemicals when incubated after myofibroblast differentiation has occurred. It has been demonstrated that myofibroblasts have the capacity to de-differentiate via stimulation of Nrf-2 [54]. Treatment with quercetin, kaempferol and capsaicin could potentially induce myofibroblast de-differentiation, because of their known effects on Nrf-2 activation [30,35,55]. Determining the optimal timing for phytochemical treatment and exploring its underlying mechanisms is essential to determine the most optimal strategy for prevention and treatment of skeletal muscle fibrosis. Alpha-SMA is widely used as a functional marker for myofibroblasts [56]. However, for measuring myoblast-to-myofibroblast differentiation in skeletal muscle, αSMA might be a suboptimal marker. While some studies show low expression or no expression of αSMA in C2C12 myoblasts, other studies show the opposite, with an increased expression found with increased differentiation status [8,37,57,58]. Therefore, we developed a method to measure myofibroblast differentiation in skeletal muscle cells. As high expression of αSMA was present in differentiated myotubes in this study, αSMA expression of MyoD-positive cells was excluded from the analysis. A mask of MyoD-expressing cells was created to measure the αSMA intensity in cells that could be either undifferentiated myoblasts or myofibroblasts. It is expected that myofibroblast cells express higher levels of αSMA compared to undifferentiated myoblasts [37].

It should be noted that our two-dimensional single-cell model, as used in this study, has limitations compared to *in vivo* skeletal muscle tissue. The lack of a three-dimensional architecture does not allow for the accumulation and distribution of ECM proteins including collagen in the surrounding environment [59]. Thus, only gene expression, and not the protein expression, of collagen type I and III were assessed in this study. A more representative *in vitro* model of skeletal muscle described by Bersini et al. comprises multiple cell types (e.g., muscle cells and endomysial muscle fibroblasts) supported by a vascular network with mural-like cells [60]. By replacing muscle fibroblasts from a healthy subject with fibroblast cells derived from a patient with Duchenne muscular dystrophy (DMD), this three-dimensional vascularized muscle tissue expressed an increased amount of ECM components collagen I and fibronectin. Moreover, DMD fibroblasts increased expression of αSMA compared to control fibroblasts, which indicates differentiation into myofibroblasts [60]. Such three-dimensional models of skeletal muscle fibrosis (in this case related to DMD) could be used to further investigate the effects of kaempferol, capsaicin and quercetin on fibrotic ECM production.

Translation of findings to human muscle requires caution. While organ fibrosis is generally associated with increased collagen content, human studies investigating alterations in skeletal muscle ECM with aging show contradicting results. One study showed that the percentage of collagen area in human skeletal muscle was significantly higher with increased age, which was mainly due to an increase in collagen type I, but no difference in collagen type III [61]. On the other hand, studies show no difference in total collagen content between the skeletal muscles of young and old individuals [62,63]. Apart from the amount of ECM proteins, the orientation and crosslinking of ECM molecules are suggested to be involved in increased skeletal muscle stiffness and decreased muscle function with increased age [64,65].

Although further validation in human models is required, this study demonstrates that quercetin, kaempferol and capsaicin can reduce myofibroblast markers such as the number of αSMA-positive cells and collagen type I/III gene expression in C2C12 cells exposed to inflammatory cytokine TGF-β1. Kaempferol emerges as the most promising of the three, showing inhibitory effects on the total expression of αSMA, both types of collagens and *Nox4* gene expression, while increasing MyoD protein and *GCLC* gene expression. These findings support the potential of these phytochemicals as anti-fibrotic treatments in skeletal muscle. Clarifying the underlying mechanisms and optimal exposure timing is critical for designing strategies to prevent or treat age-related skeletal muscle fibrosis and associated functional decline.

## 4. Materials and Methods

### 4.1. Chemicals

Bovine serum albumin (BSA), dimethylsulfoxide (DMSO), quercetin, Triton-X 100, formaldehyde solution ≥ 36%, anti-actin, α-smooth muscle mouse monoclonal antibody, anti-MyoD rabbit monoclonal antibody, goat anti-rabbit Texas-Red secondary antibody and 2-mercaptoethanol were purchased from Merck Life Sciences N.V. (Amsterdam, the Netherlands). N-2-hydroxyethylpiperazine-N-2-ethane sulfonic acid (HEPES) buffer solution (1 M), p/s (10,000 U/mL penicillin, 10,000 μg/mL streptomycin), TGF-β1, and goat anti-mouse Alexa Fluor Plus 488 secondary antibody were purchased from ThermoFisher Scientific (Waltham, MA, USA). 4′,6-diamidino-2-phenylindole (DAPI) was purchased from Abcam (Cambridge, UK). Kaempferol was purchased from TargetMol Chemicals Inc. (Boston, MA, USA). Capsaicin was purchased from Adooq Bioscience LLC (Irvine, CA, USA). iQ SYBR^®^ Green Supermix was purchased from BioRad Laboratories (Hercules, CA, USA).

### 4.2. Cell Culture and Myogenic Differentiation

C2C12 murine skeletal myoblasts (ATCC CRL-1772, Manassas, VA, USA) were cultured in growth medium containing low glucose (1 g/L) Dulbecco’s modified Eagle medium (DMEM; ThermoFisher Scientific) supplemented with 9% fetal bovine serum (FBS; ThermoFisher Scientific) (*v*/*v*) and 1% p/s (*v*/*v*) [66]. Cells were cultured in a humidified atmosphere containing 5% CO_2_ at 37 °C and passaged when reaching 60–70% confluency. Passage numbers from 10 to 15 were used for experiments. Cells were seeded on Matrigel-coated 24-well black culture plates (BIOKÉ, Leiden, The Netherlands) at a density of 12,500 cells per well (for immunohistochemistry experiments) or on 6-well clear culture plates (Greiner Bio-One, Frickenhausen, Germany) at a density of 62,500 (for qPCR and caspase experiments). After 40 h of incubation, cells were washed with Dulbecco’s phosphate-buffered saline (DPBS; ThermoFisher Scientific), and growth medium was replaced with differentiation medium containing high glucose (4.5 g/L) DMEM (ThermoFisher Scientific) supplemented with 1% heat-inactivated FBS (hiFBS, ThermoFisher Scientific) (*v*/*v*) and 25 mM HEPES (*v*/*v*) and incubated for 48 h to induce myogenic differentiation.

### 4.3. Myofibroblast Differentiation and Phytochemical Treatment

**Figure 7:**
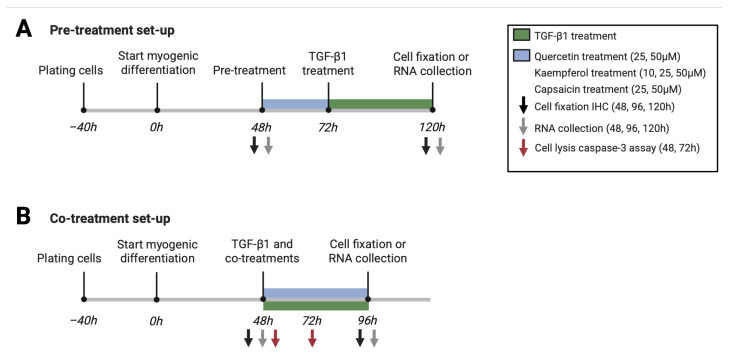
Schematic overview pre-treatment (**A**) and co-treatment (**B**) set-ups. Transforming growth factor beta 1 (TGF-β1). Created with BioRender.com (assessed on 22 May 2025).

After 48 h of myogenic differentiation, cells were exposed to 5 ng/mL TGF-β1 in high glucose DMEM containing 1 mg/mL BSA for 48 h to induce myofibroblast differentiation. Two experimental treatment set-ups were used. In pre-treatment experiments, 48 h differentiated cells were exposed to the phytochemicals 24 h before TGF-β1 treatment. In the co-treatment set-up, the phytochemicals were added simultaneously with the TGF-β1 treatment for 48 h (Figure 7). Quercetin and capsaicin were used at concentrations of 25 µM or 50 µM, whereas kaempferol was used at 10 µM, 25 µM or 50 µM. All three compounds were dissolved in DMSO, with a maximum concentration of 0.1% in the final exposure (*v*/*v*). Two control conditions were assessed (containing high glucose DMEM with 1 mg/mL BSA), that were sampled at different time points throughout the experiment. One control condition was sampled right after myogenic differentiation (control 48 h), and another control condition was sampled parallel to all other conditions (control 120 h for pre-treatment experiments and control 96 h for co-treatment experiments; Figure 7). TGF-β1 exposure was used as a positive control.

### 4.4. Immunofluorescence Stainings of Myogenic and Fibrotic Markers

Cells were fixated in 4% formaldehyde in DPBS (*v*/*v*) for 15 min. After washing in DPBS, cells were permeabilized in 0.3% Triton-X in DPBS (*v*/*v*) for 10 min and blocking was performed by 30 min incubation with 5% BSA solution in DPBS (*w*/*v*). Thereafter, cells were incubated with anti-α-smooth muscle actin mouse monoclonal antibody (4–5 µg/mL) and anti-myoblast determination protein 1 (MyoD) rabbit monoclonal antibody (0.6 µg/mL) in DPBS overnight at 4 °C. Cells were washed with DPBS and incubated with goat anti-mouse Alexa Fluor Plus 488 secondary antibody (4 µg/mL) and goat anti-rabbit Texas-Red secondary antibody (4 µg/mL) in DPBS for 45 min at room temperature followed by a DAPI nuclei staining (1.4 µg/mL in DPBS) for 15 min. An overview of antibodies used in this study is shown in Appendix A.

### 4.5. Image Analyses

Five images per well (location north, east, south, west, and center) were obtained with a fluorescent microscope (EVOS FL, ThermoFisher Scientific) and analyses were performed using Fiji software version 2.14.0/1.54f. As αSMA expression was also present in MyoD-positive myotubes, the αSMA intensity of myoblasts/myofibroblasts was assessed after masking the MyoD-positive cells using a threshold method (Auto Threshold: Triangle). MyoD intensity was measured in the total image, and nuclei were counted in the total image, in MyoD-negative cells and in MyoD-positive cells.

### 4.6. RNA Extraction and qPCR

RNA was isolated using the RNeasy Mini Kit (Qiagen N.V., Venlo, The Netherlands) according to the manufacturer’s specifications. The yield and purity of RNA were measured with the BioTek Synergy HTX plate reader (Agilent Technologies, Santa Clara, CA, USA) using the BioTek Gen5 software version 3.11. 500 ng RNA was used to synthesize complementary DNA (cDNA) with the iScript™ cDNA synthesis kit (Bio-Rad). cDNA was diluted 1:50 and quantitative polymerase chain reaction (qPCR) was performed (15 μL reaction) with the CFX Connect Real-Time PCR system (Bio-Rad) using iQ SYBR^®^ Green Supermix. Samples were incubated for 3 min at 95 °C followed by thermocycling of 12s at 95 °C and 30 s at 55 °C for 38 cycles. Primers, validated for amplification efficiency, were used at a concentration of 450 nM (Table 1). Based on the Pfaffl method [67], 14-3-3 protein zeta/delta (*YWHAZ*) and glyceraldehyde 3-phosphate dehydrogenase (*GAPDH*) were used as housekeeping genes and relative gene expression was calculated.

### 4.7. Caspase-3 Activity Assay

A colorimetric caspase-3 assay kit (Abcam) was used to assess caspase-3 activity following the manufacturer’s protocol. In brief, cells were lysed after 48 h of myogenic differentiation (control 48 h) and after 24 h of additional treatment with TGF-β1 with or without co-treatments (Figure 1). Cellular protein extracts were centrifuged for 1 min at 12,000 rpm at 4 °C. Supernatant was sampled and incubated with the substrate N-Acetyl-Asp-Glu-Val-Asp p-nitroanilide (Ac-DEVD-pNA, 200 µM) in reaction buffer containing 10 mM dithiothreitol (DTT) for 90 min at 37 °C. Immediately after substrate incubation, relative caspase-3 activity was assessed by measuring the end-point absorbance at λ = 405 nm and 37 °C using the BioTek Synergy HTX plate reader (Agilent Technologies). Caspase-3 activity was corrected for total protein content measured with a bicinchoninic acid assay (BCA, ThermoFisher Scientific). Hydrogen peroxide (H_2_O_2_) in a concentration of 50 µM was used as a positive control.

### 4.8. Statistical Analyses

Data are presented as mean ± standard error of the mean (SEM), if not normally distributed as median and interquartile range. Normality was tested with the Shapiro–Wilk test. To test for statistical significance, one-way ANOVA was used for normally distributed data (total intensity αSMA of co-treatment, qPCR data, caspase-3 activity data), and the Kruskal–Wallis test was used for not normally distributed data (all other immunofluorescence data). Statistical analyses were performed using Graphpad Prism 10.1.1 software (GraphPad Software, Boston, MA, USA) and *p*-values < 0.05 were considered statistically significant. At least three independent experiments were performed for each outcome measure. In the figure captions, N corresponds to the number of independent experiments, while *n* indicates the number of biological replicates. All data (in the text and figures) are presented as the fold change compared to control 96/120 h, except for the number of nuclei which is expressed in absolute numbers.

## Figures and Tables

**Table 1:** Overview of primers used in the study.

Gene Name	Primer Sequence Forward (5′ to 3′)	Primer Sequence Reverse (5′ to 3′)
Collagen type 1 (*Col1a1*)	CCTGGACGCCATCAAGGTCT	TTTTCCTTGGGGTTCGGGCT
Collagen type 3 (*Col3a1*)	GACCAAAAGGTGATGCTGGACAG	CAAGACCTCGTGCTCCAGTTAG
Glutamate cysteine ligase catalytic subunit (*GCLC*)	ATGTGGACACCCGATGCAGTATT	TGTCTTGCTTGTAGTCAGGATGGTTT
NADPH-oxidase 4 (*Nox4*)	GAACCCAAGTTCCAAGCTCATT	GGCACAAAGGTCCAGAAATCC
14-3-3 protein zeta/delta (*YWHAZ*)	TGCTGGTGATGACAAGAAAGGAA	AACACAGAGAAGTTGAGGGCCA
Glyceraldehyde 3-phosphate dehydrogenase (*GAPDH*)	CAACTCACTCAAGATTGTCAGCAA	TGGCAGTGATGGCATGGA

## Data Availability

The data presented in this study are openly available on DataverseNL at https://doi.org/10.34894/UYP5LY. Raw data (images) and Fiji scripts used for immunofluorescence analyses will be shared upon request.

## Abbreviations

The following abbreviations are used in this manuscript:

Ac-DEVD-pNA	N-Acetyl-Asp-Glu-Val-Asp p-nitroanilide
αSMA	alpha smooth muscle actin
BAX	BCL-2-like protein
β-catenin	catenin beta-1
BCL-2	B-cell lymphoma 2
BSA	bovine serum albumin
cDNA	complementary DNA
Col1a1	collagen type 1
Col3a1	collagen type 3
DAPI	4′,6-diamidino-2-phenylindole
DMD	Duchenne muscular dystrophy
DMEM	Dulbecco’s modified Eagle medium
DMSO	dimethylsulfoxide
DPBS	Dulbecco’s phosphate-buffered saline
DTT	dithiothreitol
ECM	extracellular matrix
FBS	fetal bovine serum
GAPDH	glyceraldehyde 3-phosphate dehydrogenase
GCLC	glutamate cysteine ligase catalytic subunit
GSH	glutathione
HEPES	N-2-hydroxyethylpiperazine-N-2-ethane sulfonic acid
hiFBS	heat-inactivated fetal bovine serum
H_2_O_2_	hydrogen peroxide
IGF1R/AKT/mTOR	insulin-like growth factor 1/protein kinase B/mammalian target of rapamycin
ITG1B/FAK/paxillin	integrin-β1/focal adhesion kinase/paxillin
LDH	lactate dehydrogenase
MAPK	mitogen-activated protein kinase
mTOR	mammalian target of rapamycin
MyoD	myoblast determination protein 1
MyoD(-)	myoblast determination protein 1-negative
MyoD(+)	myoblast determination protein 1-positive
NADPH oxidase/Nox	nicotinamide adenine dinucleotide phosphate oxidase
Nrf-2	nuclear factor erythroid 2-related factor 2
qPCR	quantitative polymerase chain reaction
ROS	reactive oxygen species
SEM	standard error of the mean
TGF-β1	transforming growth factor beta 1
YWHAZ	14-3-3 protein zeta/delta

## Appendix A

**Table A1:** Overview of primary and secondary antibodies used in the study.

Antigen	Host Species	Species Reactivity	Dilution Factor	Supplier	Category Number
α-Smooth muscle actin	Mouse	Mouse, human, rat, chicken, frog, canine, rabbit, guinea pig, goat, bovine, sheep, snake	1:500	Sigma Aldrich (Saint Louis, MO, USA)	A5228
MyoD	Rabbit	Mouse, human	1:500	Sigma Aldrich	ZRB1452
	Host species	Species reactivity	Dilution factor	Supplier	Category number
Alexa Fluor™ Plus 488	Goat	Mouse	1:500	ThermoFisher Scientific (Waltham, MA, USA)	A48286
Texas Red	Goat	Rabbit	1:500	Sigma Aldrich	SAB3700888
